# Humoral and cellular response to SARS-CoV-2 BNT162b2 mRNA vaccine in hemodialysis patients

**DOI:** 10.1186/s12865-021-00458-0

**Published:** 2021-10-19

**Authors:** Jan Melin, Maria K. Svensson, Bo Albinsson, Ola Winqvist, Karlis Pauksens

**Affiliations:** 1 Department of Medical Sciences, Renal Medicine, Uppsala University Hospital, Uppsala University, 751 85 Uppsala, Sweden; 2 Department of Medical Biochemistry and Microbiology, Zoonosis Science Centre, Uppsala University, Uppsala, Sweden; 3 Laboratory of Clinical Microbiology, Uppsala, Sweden; 4 Department of Clinical Immunology, Karolinska University Hospital, Stockholm, Sweden; 5 ABC Labs, Solna, Sweden; 6 Department of Medical Sciences, Infectious Medicine, Uppsala University, Uppsala, Sweden

**Keywords:** Hemodialysis, Covid-19, Covid-19 spike protein, Vaccination, Humoral response, Cellular response

## Abstract

**Background:**

Hemodialysis (HD) patients have an increased risk of acquiring infections due to many health care contacts and may, in addition, have a suboptimal response to vaccination and a high mortality from Covid-19 infection.

**Methods:**

In 50 HD patients (mean age 69.4 years, 62% men) administration of SARS-CoV-2BNT162b2 mRNA vaccine began in Dec 2020 and the immune response was evaluated 7–15 weeks after the last dose. Levels of Covid-19 (SARS-CoV-2) IgG antibody against the nucleocapsid antigen (anti-N) and the Spike antigen (anti-S) and T-cell reactivity testing against the Spike protein using ELISPOT technology were evaluated.

**Results:**

Out of 50 patients, anti-S IgG antibodies indicating a vaccine effect or previous Covid-19 infection, were detected in 37 (74%), 5 (10%) had a borderline response and 8 (16%) were negative after two doses of vaccine. T-cell responses were detected in 29 (58%). Of the 37 patients with anti-S antibodies, 25 (68%) had a measurable T-cell response. 2 (40%) out of 5 patients with borderline anti-S and 2 (25%) without anti-S had a concomitant T-cell response. Twenty-seven (54%) had both an antibody and T-cell response. IgG antibodies to anti-N indicating a previous Covid-19 disease were detected in 7 (14%) patients.

**Conclusions:**

Most HD patients develop a B- and/or T-cell response after vaccination against Covid-19 but approx. 20% had a limited immunological response. T-cell reactivity against Covid-19 was only present in a few of the anti-S antibody negative patients.

## Background

The immune system is affected by uremia. The impact on the immune system of uremia has been described as “uremia associated immunological aging” and it has been suggested the T-cell system is aged 15–20 years compared to a healthy individual of the same age [1]. Uremic toxins seem to have inhibitory effects on immune cells [2] and it has been shown that this may decrease the efficacy of vaccines [1]. Patients with end stage kidney disease (ESKD) treated with in-center hemodialysis (HD) have been a vulnerable group during the Covid-19 (Coronavirus disease 2019) pandemic [3]. HD patients cannot isolate themselves due to in-clinic dialysis several days a week. In addition, HD patients also have other health issues that require health care visits. Several studies have shown an increased risk for patients on dialysis to be infected by covid-19 [4, 5] and data from the ERA-EDTA (European Renal Association—European Dialysis and Transplant Association) registry showed a 20% mortality risk in dialysis patients infected by Covid-19 during the spring of 2020 [3]. The combination of being at an increased risk of acquiring Covid-19 infection and at the same time being at a high risk of severe morbidity and mortality if infected makes protective measures such as vaccination important in HD patients.

According to a recent review most HD patients develop an antibody response to the SARS-CoV-2 (Severe Acute Respiratory Syndrome Coronavirus 2) BNT162b2 mRNA vaccine [6]. However, the response rate has varied considerably between studies, the highest response rate, 96.4%, was found by Grupper et al. [7] and the lowest, 72.8%, by Simon et al. [8]. Previous studies also showed that while most HD patients developed antibodies against Covid-19 the response was attenuated, and a significant proportion of the patients failed to produce measurable antibody levels against Covid-19 after one or two doses of vaccination.

There are few studies that have addressed the cellular response to SARS-CoV-2 BNT162b2 mRNA vaccine in HD-patients [9–13]. In the ROMANOV study a decreased cellular response to the BNT162b2 vaccine was found [9]. In addition, three recent studies showed that the cellular response to the vaccine was slightly decreased in dialysis patients but to a lesser extent than in transplanted patients on triple immunosuppression therapy [10–12]. In contrast, in a small study with seven transplanted patients without humoral response to SARS-CoV-2 BNT162b2 mRNA vaccine all patients developed a cellular response [14]. This could be in line with Braun et al. that found that 35 percent of healthy blood donors without a history of Covid-19 or vaccination have T-cell reactivity to SARS-CoV-2 suggesting a potential cross reactivity to other corona viruses [15].

The question whether a cellular response to the vaccination could compensate for the attenuated antibody response in dialysis patients is not resolved and warrants further exploration and the aim of this study was to investigate the humoral and cellular response to the SARS-CoV-2 BNT162b2 mRNA vaccine in a Swedish cohort of hemodialysis patients.

## Results

### Patients

A total of 50 patients signed informed consent and were included in the study. Clinical and demographical characteristics of study participants (n = 50) are shown in Table 1. The mean age was 69.4 years and 62% were men. Dialysis vintage was 81 ± 19 months but the range was wide, 5–470 months.

**Table 1:** Demographic data and clinical characteristics of all patients included in the study (n = 50)

Age (years)	69.4 ± 14.1 (25–90)
Men/women	31/19, 62 vs 38%
Body Mass Index (BMI, kg/m^2^)	26.1 ± 5.6 (13–44)
Dialysis (months)	65.1 ± 74.0 (5–470)
Diabetes (n, %)	23 (46)
Nephrosclerosis (n, %)	15 (30)
Autosomal Dominant Polycystic Disease (n, %)	4 (8)
Chronic glomerulonephritis (n, %)	8 (16)
Vasculitis/anti-GBM-nephropathy (n, %)	4 (8)
Ongoing medication with CNIs (n, %)	2 (4)
Ongoing medication with MMF (n, %)	1 (2)
Previous^a^ treatment with CNI (n, %)	3 (6)
Previous^a^ treatment with Rituximab or Cyclophosphamide (n, %)	5 (10)
Current systemic steroid treatment (n, %)	7 (14)
Previous renal transplant (n, %)	5 (10)

Results are expressed as mean ± SD and range (min, max) or as proportions *n* (%)

CNI, calcineurin inhibitors; MMF, mycophenolate mofetil; GBM, glomerular basement membrane

^a^Within the last ten years

### Antibodies against the Spike-protein (anti-S)

**Fig. 1:**
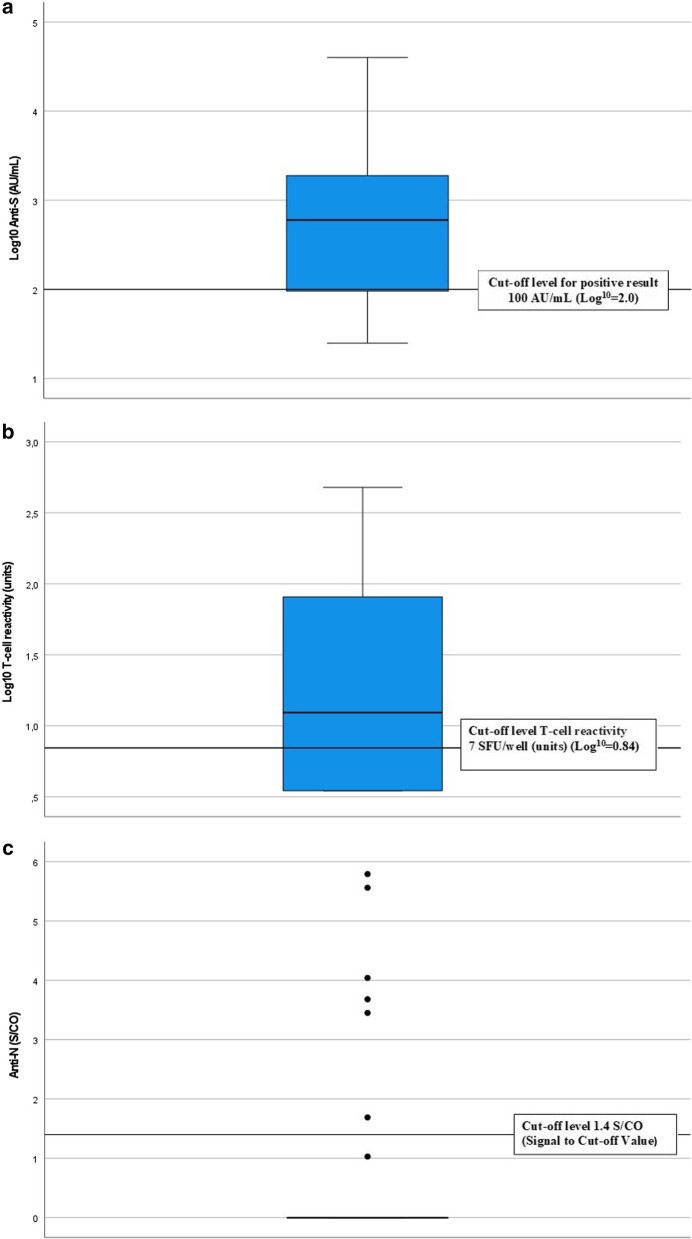
Immunoreactivity after SARS-CoV-2 BNT162b2 mRNA vaccine in HD-patients. **a** IgG-antibody response to Covid-19 spike protein (Log10 anti-S). The cut-off for positive results was set to 100 AU/mL (Abbott Units/milliliter) and 50 AU/mL for negative results (quantitative method). Values between 50 and 100 AU/mL were considered borderline. **b** T-cell reactivity to Covid-19 spike protein (Log10). The cut off of the ELISPOT assay was set to 7 SFU/well (units). **c** IgG-antibody response to Covid-19 nucleocapsid antigen (anti-N). The cut-off for the qualitative method for anti-N was 1.4 S/CO (Signal to Cut-off Value). Values between 0.9 and 1.39 were consider borderline

The distribution of antibodies against the Spike-protein is shown in Fig. 1a. A value below 50 AU/mL (7.1 BAU/mL) was considered negative and values between 50 and 99 (7.1–14.2 BAU/mL) AU/mL borderline. Thirty-seven patients (74%) displayed significant antibody levels to the Spike-protein, five patients (10%) had a borderline response, and eight (16%) were negative. Anti-S levels against the Spike-protein showed a correlation to age but none of the other variables (Table 2). Patients without anti-S response were more likely to have history of any previous or current immunosuppressive therapy (n = 2) of any kind (calcineurin inhibitors, rituximab, azathioprine, mycophenolate mofetil, or cyclophosphamide) than those who had never been treated with immunosuppressants (*p* < 0.05).

**Table 2:** Correlations between immunological responses and patient characteristics

	Anti-N (s/co)		Anti-S (AU/mL)		T-cell reactivity (units)	
	Spearman’s rho	*P* value	Spearman’s rho	*P* value	Spearman’s rho	*P* value
Age (years)	− 0.123	0.395	− 0.292	0.040	− 0.234	0.105
BMI (kg/m^2^)	0.073	0.614	− 0.162	0.262	− 0.021	0.883
Dialysis (months)	− 0.059	0.682	− 0.017	0.906	− 0.118	0.413
S-Albumin (g/L)	− 0.238	0.100	− 0.167	0.253	0.030	0.836
Urea before dialysis (mmol/L)	− 0.069	0.635	0.048	0.740	0.274	0.054
Anti-N (s/co)	–	–	0.490	< 0.001	0.451	< 0.01
Anti-S (AU/mL)	0.710	< 0.001	–	–	0.481	< 0.001
T-cell react (units)	0.443	0.001	0.564	< 0.001	–	–

anti-S, IgG-antibody response to Covid-19 Spike protein; anti-N, IgG-antibody response to Covid-19 nucleocapsid antigen; S/CO, Signal to Cutoff Value; AU/mL, Abbott Units/milliliter

### Cellular reactivity against Covid-19

**Fig. 2:**
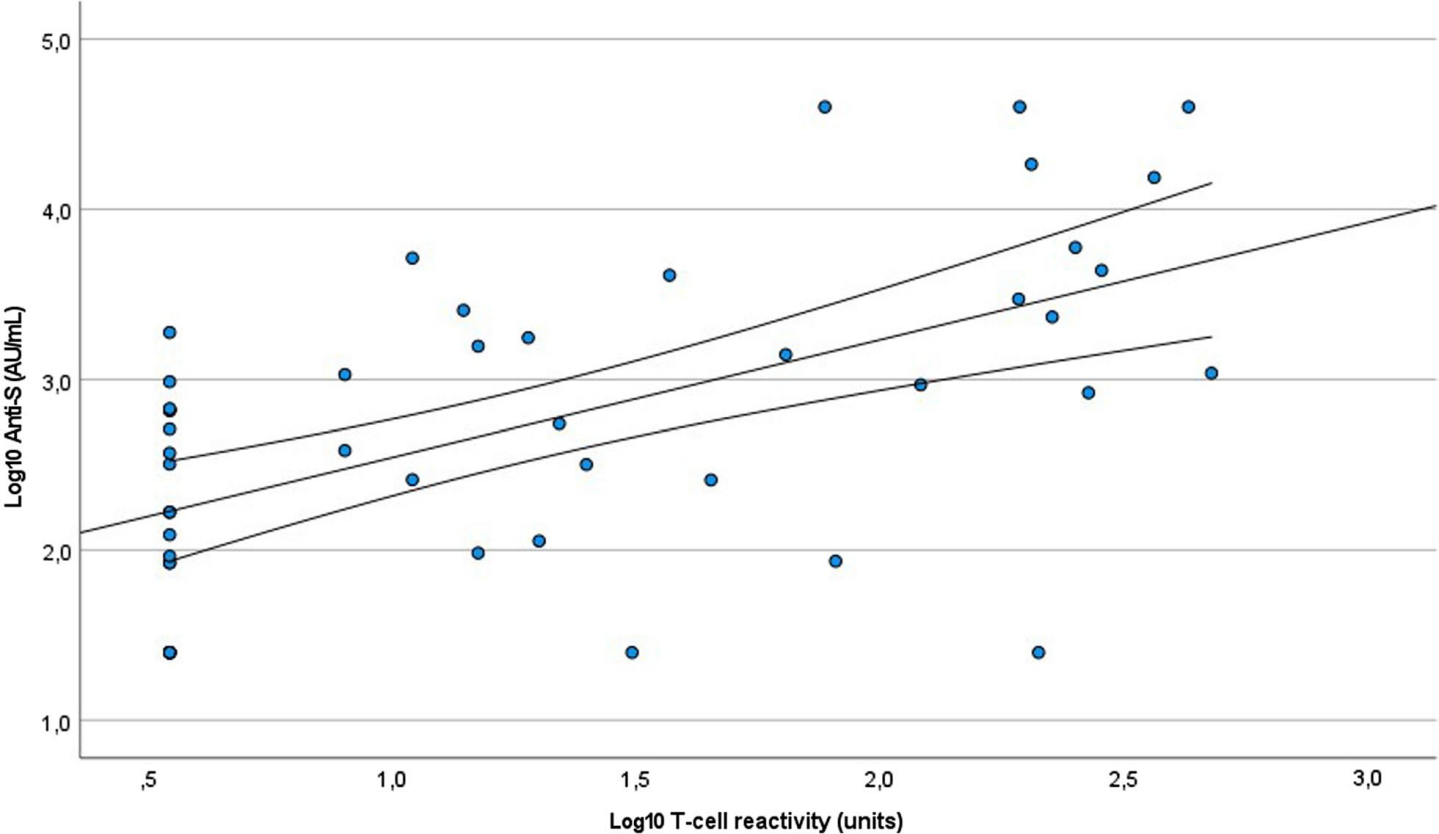
Correlation between IgG-antibody response to Covid-19 spike protein (Log10 anti-S) and T-cell reactivity to Covid-19 spike protein (Log10 T-cell reactivity)

The distribution of T-cell reactivity against the Covid-19 spike protein is shown in Fig. 1b. Values below seven (7) units were considered negative. A total of 29 patients (58%) showed cellular reactivity against Covid-19 spike protein after vaccination with BNT162b2 mRNA vaccine. No significant association was found between T-cell reactivity and the variables in Table 2, but it is worth mentioning that a near-significant correlation was found between t-cell reactivity and the mean urea level before dialysis during the study period (*p* = 0.054) and that there was a tendency that patients with diabetes (n = 23) more often had a negative T-cell response (*p* = 0.085). Interestingly, two of the patients who were negative for anti-S antibodies and two with borderline response had a positive T-cell response. Nine of the patients in the study had a weak both humoral and cellular response to the vaccine. There was a significant correlation between anti-S levels and T-cell response (rho = 0.546, *p* < 0.001) (Table 2; Fig. 2). All patients who previously had tested positive for Covid-19 by PCR-test (see below) had T-cell reactivity against the Covid-19 spike protein.

### PCR-positive Covid-19 infections

Three patients (6%) had tested positive for Covid-19 by PCR before the vaccination commenced. Two patients (4%) became PCR-positive between doses one and two. One of these patients was admitted to the hospital but did not need intensive care. The other patients were not hospitalized. After dose two, 4 patients (8%) had PCR-positive tests and all of them developed a mild disease. None of the patients that infected after two doses of vaccination were admitted to the hospital.

### Antibodies against the nucleocapsid antigen (anti-N)

The distribution of antibodies against the nucleocapsid antigen (anti-N) is shown in Fig. 1c. There was a cut off where values below 0.9 s/co were considered negative. Seven patients (14%) had a positive immune response to anti-N indicating a previous Covid-19 infection. Of these, six had previously had a positive PCR-test for Covid-19. One patient had a positive response to anti-N despite never having tested positive for Covid-19. Three patients with a positive PCR-test for Covid-19 were negative for anti-N. Anti-N did not correlate with any of the variables in Table 2.

## Discussion

In this study we show that the majority of patients with end-stage kidney disease (ESKD) and hemodialysis treatment (HD) developed a B- and/or T-cell response after vaccination against Covid-19 but approx. one-fifth (20%) had a limited immunological response. This study is in line with previous published data that most, but not all, HD patients develop a serologic antibody response to Covid-19 vaccination [6] and the proportion of HD patients that responds to the vaccine is much higher than in transplanted patients [10]. However, a significant proportion of the patients in our study has no or a very limited immune response to the vaccine. Broseta and co-workers have shown that in HD patients with a serological response the vaccination displayed a full effect six weeks after the first dose of vaccine [16]. In our study the elapsed time between the second dose and the blood sampling is at least seven weeks and thus the timing between vaccination and assessment is not likely to explain non-response.

The most interesting finding in our study is that more than 40% of the HD patients did not develop a cellular response to the spike protein of SARS-Cov-2. In addition, only two of the patients without a serological response displayed a cellular response. Our interpretation is that in our study cellular response adds little to measurable protection against Covid-19. Our results are in contrast with the findings from other previous studies, where the cellular response was over 90% (10–12) but are more in line with the ROMANOV-study (9) and Broseta and co-workers (13). The reason for this difference is not clear. Even though age might be a factor in the immune response it is hardly the sole explanation. In our patient cohort the mean age was 69.4 years, in the ROMANOV-study [9] 64.9, and in the three other studies, 67.6, 67.4, 71.2 and 68.6 years, respectively [10–13]. Perhaps differences in the assessment of the cellular response thus may the most important factor to consider and warrants further evaluate.

Due to the small sample size and lack of power, it was difficult to find factors associated with an attenuated vaccine response. However, in this study we found a negative correlation between age and the humoral response to the vaccine such that older age was associated with an attenuated response. A similar effect has been shown in other studies [17]. There was also a higher frequency of previous or current use of immunosuppressant drugs in among the non-responders. In this cohort, only two patients, were on current and ongoing immunosuppression so the association found could also be a marker for type of underlying disease for kidney failure and previous kidney transplantation.

The finding that a significant proportion of the patients on hemodialysis do not have any measurable protection against Covid-19 may have clinical implications. Precautions to stop spreading the virus in dialysis units such as wearing of masks, isolation of patients with symptoms of upper respiratory before testing and restriction regarding visitors should be continued. This finding also raises the question of extra vaccine doses. In a study of the impact of a third dose of BNT162b2 mRNA vaccine in HD patients five out of 12 patients who were antibody negative after two doses seroconverted after a third dose [18].

This study has several limitations. The number of patients is small and the power to detect associations between clinical characteristics and response to vaccination therefore limited. Since this study was designed after the vaccination started, we do not have any data on antibody levels and T-cell reactivity before the vaccination. Our HD patients were among the first to be vaccinated and thus it was not possible to find a suitable control group of vaccinated at the same time. However as mentioned in the methods section, the average response from healthy individuals after two vaccine doses was 58 SFU/well in data from ABC-labs. Another limitation is the wide range in time between vaccination and assessment of immune response due to the fact that all patients were not vaccinated at the same time. In addition, a considerable proportion of the patients in this study were infected with Covid-19 either before, during or after the vaccination and makes the effect of the vaccination less clear. The strength of this study is that it is done in a relevant real-world clinical setting and that the patient’s immune response to the vaccination now will be followed over time.

## Conclusions

In conclusion, a considerable portion of the HD patients does not have protection against Covid-19 by antibodies and few of the antibody negative patients have a cellular response to Covid-19 after two doses of SARS-CoV-2 BNT162b2 mRNA Vaccine. This must be taken into account and considered when caring for patients in the dialysis units and warrants further research regarding the immune response after vaccination in these patients.

## Methods

### Patients

Fifty incident dialysis patients in two dialysis units at Uppsala Academic Hospital, Sweden, were vaccinated with the SARS-CoV-2 BNT162b2 mRNA vaccine (PfizerBionTech). The patients in the study received their first vaccine dose from 28 December 2020 to 22 January 2021 and the second dose from 20 January 2021 to 10 March 2021. The patients were tested for their immune response during late April and early May, seven to 15 weeks after their last vaccine dose. Blood samples for analyses of immune response were drawn from the patients prior to the start of dialysis.

### Analysis of SARS-CoV-2 IgG antibodies

SARS-CoV-2 IgG antibodies were analyzed at the laboratory of clinical microbiology, Uppsala university hospital, using both SARS-CoV-2 IgG II Quant assay for quantitative determination of IgG antibodies to SARS-CoV-2 (spike receptor-binding domain/anti-S) and SARS-CoV-2 IgG for qualitative determination of IgG antibodies to SARS-CoV-2 (nucleocapsid domain/anti-N). Both analyzed on Abbott Architect i2000SR Analyzer (Abbott, Illinois, USA). The cut-off was set to 100 AU/mL (Abbott Units/milliliter) for positive result for the quantitative method and 1,4 S/CO (Signal to Cutoff Value) for the qualitative method.

### Enzyme-linked immunospot—EliSPOT

The analyses of T-cell reactivity were performed at ABC-labs, Solna Sweden https://www.abclabs.se/. Peripheral blood mononuclear cells (PBMC) were purified from heparinized whole blood by centrifugation at 800×*g* for 20 min in pluriMate tubes (Pluriselect), followed by washing with Phosphate Buffer Saline pH 7,4 pH, and centrifugation at 450×*g* for 8 min, repeated 3 times. Duplicates of 250 000 cells/well were plated in AIM V media (Glibco) duplicate onto pre-coated strip plates with monoclonal antibodies for IFN-g (Human IFN-g SARS-CoV-2 ELISPOTplus kit, MabTech, Stockholm Sweden).

Cells were stimulated with a pool of 81 synthesized peptides covering the spike 1 protein from SARS-CoV-2, or anti-CD3 and anti-CD-28 antibodies as positive control (MabTech). After 16 h of incubation at 37 °C, IFN-g production was detected by incubation with biotinylated anti IFN-g antibodies followed by streptavidin conjugated with alkaline phosphatase, according to instructions. Reactions were developed with BCIP/NBT substrate and analyzed using the MabTech Astor ELISpot reader.

The cut off of the ELISPOT assay was set to 7 SFU/well, with a sensitivity of 87% and a specificity of 98% based on samples from symptomatic SARS-CoV-2 PCR positive individuals and samples collected pre-covid. True positive samples responded with 119.8 ± 198.8 SFU/well (Mean ± SD) and true negative samples with 5.4 ± 2.8 SFU/well (Mean ± SD). In comparison average response from healthy individuals after 1 vaccine dose was 31.1 SFU/well whereas the average response in samples after two vaccine doses was 58 SFU/well. (Data from ABC-labs, Solna Sweden).

### Statistics

Descriptive statistics was used to describe demographical and clinical characteristics in Table 1. Means with standard deviations and range were used to describe quantitative variables. Absolute frequencies and percentages were used for categorical variables. Differences between groups were assessed with Fischer’s exact test. Spearman's Rank correlation was used to compare the relationship between variables. Anti-S and T-cell reactivity in the graphs were log-transformed to log10. Anti-S or T-cell reactivity below the detection levels < 50 AU/mL and < 7 units was set to half the detection level in this analysis, i.e., 25 AU/mL and 3.5 units, respectively. Statistical analyses were performed with IBM SPSS Statistics version 28 software (IBM, Armonk, NY, USA).

## Data Availability

All data in this paper can be obtained from the corresponding author by request.

## Abbreviations

anti-N: IgG antibodies to SARS-CoV-2 nucleocapsid domain
anti-S: IgG antibodies to SARS-CoV-2 spike receptor-binding domain
AU/mL: Abbott Units/milliliter
AZA: Azathioprine
BAU/mL: Binding Antibody Units/mL
BCIP/NBT: 5-Bromo-4-Chloro-3-Indoly-Phosphate/NitroBlue Tetrazolium chloride
BMI: Body Mass Index
CNI: Calcineurin inhibitors
Covid-19: Coronavirus disease 2019
DM: Diabetes mellitus
EliSPOT: Enzyme-Linked ImmunoSpot
ERA-EDTA: European Renal Association—European Dialysis and Transplant Association
ESKD: End-stage kidney disease
GBM: Glomerular Basement Membrane
HD: Hemodialysis
MMF: Mycophenolate Mofetil
SARS-CoV-2: Severe Acute Respiratory Syndrome Coronavirus 2)
S/CO: Signal to Cutoff Value
